# Isolation and Identification of *Acholeplasma* sp. from the Mud Crab, *Scylla serrata*

**DOI:** 10.1155/2011/209406

**Published:** 2011-07-17

**Authors:** Ji-Gang Chen, Dan Lou, Ji-Fang Yang

**Affiliations:** ^1^Municipal Key Laboratory of Microorganism and Environmental Engineering, Ningbo, Zhejiang 315100, China; ^2^College of Biological and Environmental Sciences, Zhejiang Wanli University, Ningbo, Zhejiang 315100, China; ^3^College of Food Science and Technology, Huazhong Agricultural University, Wuhan, Hubei 430070, China

## Abstract

For the first time, a mollicute-like organism (MLO) was cultured from moribund mud crabs (*Scylla serrata*) during an outbreak of clearwater disease in Zhejiang Province, China. The MLO displayed a fried-egg colony morphology in culture, did not possess a cell wall, and was not retained by 0.45 *μ*m and 0.2 *μ*m filters. It was able to ferment glucose, sucrose, lactose, and maltose, but it did not utilize arginine and urea. The MLO grew in the absence of bovine serum and was not susceptible to digitonin. Sequence analysis of the 16S rRNA gene revealed that this MLO had 99% identity with *Acholeplasma laidlawii* PG-8A, which indicates that the organism isolated from mud crabs is a member of the genus *Acholeplasma*.

## 1. Introduction

The class Mollicutes represents a unique category of bacteria, the members of which are characterized by a small cell size, the absence of a cell wall, a reduced genome, and a simplified metabolic pathway [1]. They can be pathogenic or saprophytic and commensal [2]. To date, mollicutes have been observed and identified in many vertebrate, insect, and plant hosts [2]. Mollicutes also have been reported from several aquatic animals, such as fish [3], shrimp [4–8], crab [9], oyster [10], crayfish [11, 12], and bryozoan [13, 14]. However, mollicutes of aquatic animals, especially those of crustaceans, have not been studied extensively. Only a few mollicutes associated with crustaceans have been isolated, purified, and had their taxonomic status confirmed [4, 9]. 

The mud crab, *Scylla serrata *(Forska), traditionally called the green crab, is an economically important marine species cultured in the Chinese provinces of Zhejiang, Fujian, Guangdong, Guangxi, and Hainan. Since the 1990s, the *S. serrata* aquaculture industry has experienced rapid growth. However, the industry also is facing increasing economic losses caused by the outbreak of various diseases, such as sleeping disease (SD) [15] and milk disease [16]. In 2005, an epidemic of clearwater disease (CD) broke out in Zhejiang Province. The symptoms of this disease included debility, weak grip strength of pincers, hydroabdomen, white carapace, drying of gill filaments, and weak blood coagulation capacity. The estimated mortality at the affected farms was ~80%. Mollicute-like organisms (MLOs) together with reo-like viruses (unpublished data) have been implicated as causes of CD. However, the MLO has not been isolated and cultivated, thus the precise taxonomic status and pathogenesis of the MLO in *S. serrata* have been unclear. 

In this study, the MLO from mud crabs showing signs of CD was isolated and cultivated. The taxonomic classification of this organism was determined by morphology, physiological properties, and DNA analysis, and its pathogenesis was investigated. 

## 2. Materials and Methods

### 2.1. Mud Crab

Two male and three female moribund or dead mud crabs with CD were obtained from a pond of a mud crab farm during the CD outbreak in August 2005 in Sanmen County, Zhejiang Province. Using electron microscopy, two different organisms were detected in the five crabs: reo-like viruses and MLOs (unpublished data). 

### 2.2. Culture

In a previous study, we found that the MLO was present mainly in the epithelium of gill cells (unpublished data). Therefore, the gill was selected for isolation of the MLO. Excised gill tissue was placed in mycoplasma liquid medium (MLM); each 100 mL of medium contained 2.55 g of mycoplasma broth base (Frey), 0.5 mL of 0.4% phenol red, 0.2 mL of 10% thallium acetate (Sigma), 20 mL of mycoplasma-free FBS (Hangzhou Sijiqing Biological Engineering Materials Co., Ltd.), 1 mL of freshly prepared yeast extract solution, 1 mL of ampicillin solution (10 mg mL^−1^), and 1 mL of 10% glucose solution, and the solution was adjusted to pH 7.8. MLO solid medium (MSM) was prepared in the same manner as described above, but it contained 1% medium technical agar (Oxoid). It was supplemented with mycoplasma broth base, yeast extract solution, FBS, thallium acetate, and ampicillin in the same concentrations as those used for MLM, but it did not contain phenol red and glucose.

The MLO culture procedure was designed as previously described by Ghadersohi and Owens [4] with slight modification. Briefly, gill and gut tissue from individual crabs was homogenized in 3 mL of MLM at 4°C using a glass tissue blender. Once a homogenous suspension was produced, 200 *μ*L aliquots were used to prepare a series of 10-fold dilutions in MLM. Negative controls consisted of FBS and other medium constituents in MLM. Inoculated tubes were incubated at 37°C and examined daily for pH (color) changes. Whenever the color of the medium turned from red to yellow, 300 *μ*L of the culture medium were transferred into a tube containing 3 mL fresh of MLM. After 6 to 7 days, 50 *μ*L from each tube with the highest dilution indicating growth was spotted onto MSM plates. The plates were incubated in a humidified atmosphere with 5% CO_2_ at 37°C for 14 days. Inoculated plates were examined for the presence of colonies using a stereomicroscope (Olympus). MLO colony growth differences in plates incubated aerobically and in 5% CO_2_ were recorded. Cellular morphology of the organisms was examined by light microscopy after application of Gram and Giemsa stains. 

### 2.3. Colony Staining

To observe MLO colonies and differentiate between *Mollicute* and bacterial L-form colonies, MLO colonies were stained with Dienes stain [17] as described by Ghadersohi and Owens [4]. The preparation was then examined with a microscope under low power. 

### 2.4. Purification Experiment

Isolated MLOs were purified using the single colony technique [4]. A single colony was removed by cutting out a small block of agar using a sterile scalpel. The colony was transferred into a tube containing 3 mL of MLM and incubated for 48 h. The culture was diluted 1/10 and 1/100 in MLM, and 50 *μ*L of each dilution were spotted onto MSM plates and incubated in a humidified atmosphere containing 5% CO_2_ at 37°C for 7 days. This purification procedure was repeated three times. 

### 2.5. Ultramicroscopy

For ultrathin sectioning, MLOs on MLM medium were pelleted by centrifugation (12 000 g for 10 min at 4°C), resuspended in 2.5% glutaraldehyde, embedded in 4% Noble agar, placed on Formvar-coated copper grids for solidification, and fixed again in 2.5% glutaraldehyde in phosphate buffered saline (PBS; 0.1 mol L^−1^, pH 7.2) at 4°C for 2 h. After several rinses with PBS, the samples were post-fixed with 1% OsO_4_ for 1 h. Subsequently, the tissues were dehydrated in an ethanol series and embedded in Spurr's resin. Ultrathin sections were stained with uranyl acetate and lead citrate and observed under a transmission electron microscope (TEM). 

### 2.6. Biochemical Tests

The mud crab MLO's metabolism of glucose, sucrose, lactose, and maltose [18, 19] was examined, as was its hydrolysis of arginine and urea [20, 21] and its reduction of tetrazolium chloride and methylene blue [4]. All plates and tests were incubated at 37°C in a humidified atmosphere with 5% CO_2_ for 7 days. 

### 2.7. Sterol Requirement

The MLO's sterol requirement was established by testing the susceptibility of the isolates to digitonin and by placing the isolates in an MLM lacking serum [22].

### 2.8. Haemolysis and Hemadsorption

The isolated MLO was examined for hemolytic activity and hemadsorption using sheep, chicken, and rabbit erythrocytes using previously described methods [23]. 

### 2.9. Filtration Studies

MLO cultures were diluted 1 : 10 in a liquid medium and filtered through membrane filters (Millipore) with pore diameters of 0.22 *μ*m and 0.45 *μ*m. The numbers of colony-forming unit (CFU) per milliliter in the filtrates were determined by plating the filtrates onto agar and were compared with the numbers of CFU per milliliter in an unfiltered culture dilution [24]. 

### 2.10. Reversion Experiment

Isolated MLOs were subcultured eight consecutive times in liquid or solid growth medium lacking ampicillin or thallium acetate to determine whether the organisms reverted to bacterial L forms. Agar plates and fluid cultures of all passages were examined for alterations in the morphology of clones and cells, respectively. In addition, the agar colonies of each clone were stained with Dienes stain and examined with low power light microscopy. 

### 2.11. Analysis of Partial 16S rRNA Gene Sequence

DNA for phylogenetic analysis was extracted from mid-log phase cultures after five passages of a clonal MLO isolate (strain ZJ2005) using the QIAamp DNA Mini kit (Qiagen). The 16S rRNA gene was amplified using M1 and M2 primers [24], cloned into the pMD18-T vector (TaKaRa), and then transformed into *E. coli *Top 10 competent cells. Plasmid DNA, which was purified using the QIAprep Spin Miniprep kit (Qiagen), was sequenced afterwards. The obtained 16S rRNA gene was compared to archived genetic sequences using BLAST searches within the GenBank database [25]. Highly similar sequences were selected for phylogenetic tree construction. The phylogenetic tree was constructed with the neighbor-joining method using MEGA 4.1 software [26]. 

### 2.12. Experimental Infection

The pathogenesis of ZJ2005 was tested in a mud crab bioassay. ZJ2005 cultures were grown in 5 mL of MLM at 37°C for 48 h, after which a decimal dilution series was made in 1 mL MLM. An aliquot from each dilution was spotted onto MSM. The number of colonies on the agar was used to calculate the number of ZJ2005 organisms in the MLM culture. 

A total of 30 clinically healthy mud crabs from a research breeding facility were used in the experimental infections and randomly placed into one of three groups. Members of Group 1 (*n* = 10) were injected with 200 *μ*L of 0.75% saline water containing 1 × 10^6^ CFU ZJ2005 into a leg joint of the fifth pair of pereiopods; the crabs then were placed in a 20‰  salinity, pathogen-free, 30 L aquarium and held at 25–28°C. Members of Group 2 (*n* = 10) were exposed to ZJ2005 by bathing them in 10 L of aerated sea water (20‰  salinity) in aquaria containing 1 × 10^6^ CFU ZJ2005 at 28°C for 4 h. These passively exposed crabs were removed and placed in another 20‰  salinity, pathogen-free, 30 L aquarium at 25–28°C. Group 3 (*n* = 10) acted as the control group; crabs in this group were injected with 200 *μ*L of sterile 0.75% saline water and then held in a 20‰  salinity, pathogen-free, 30 L aquarium at 25–28°C. 

## 3. Results

### 3.1. Cultivation of Clinical Samples

**Figure 1:**
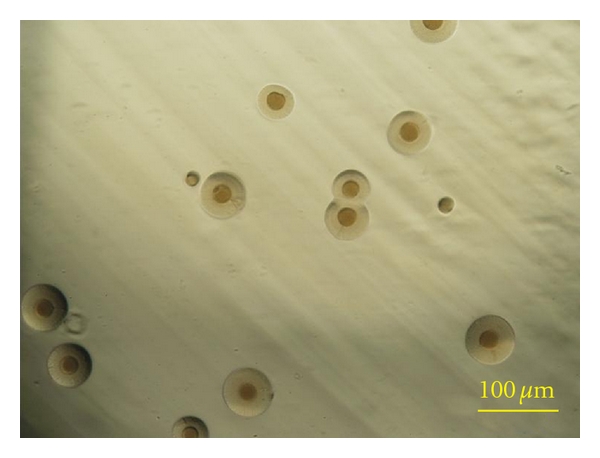
Typical fried-egg colony morphology of the mollicute-like organism from *Scylla serrata*. It was cultured on mycoplasma liquid medium under aerobic conditions for 5 days (bar = 100 *μ*m).

MLOs were removed from all moribund mud crabs (*n* = 5). Isolated and cultured MLOs decreased the pH of the MLM and formed typical fried-egg shaped colonies (Figure 1). The colonies were readily stained with Dienes reagent, which confirmed that the isolate was a true member of the Mollicutes rather than a bacterial L form [4]. 

### 3.2. Morphology

**Figure 2:**
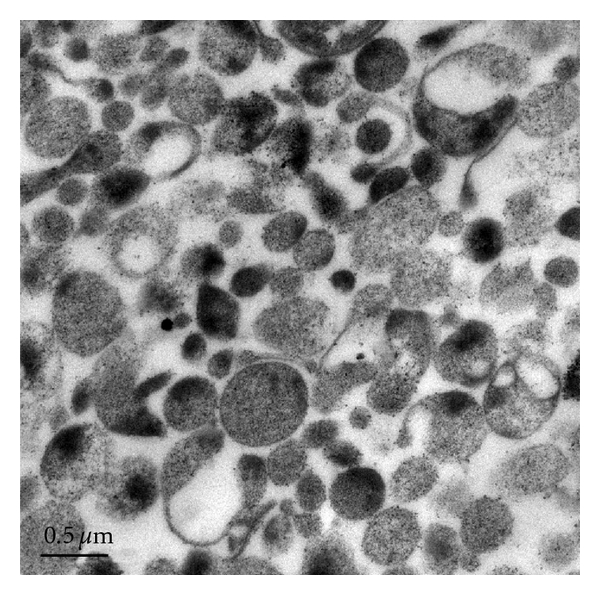
Electron micrograph of an ultrathin section of the mollicute-like organism from *Scylla serrata* (bar = 0.5 *μ*m).

Ultrathin sections showed two morphological types of cells: (i) markedly electron-dense filamentous lobulated cells of various shapes, but often they were curved (0.5–2 *μ*m) and (ii) considerably larger cells (0.1–0.5 *μ*m) of a more oval shape with a less compact and a less dense cytoplasm (Figure 2). The cells were bounded by a single unit membrane and contained densely packed ribosomes, between which were found fine strands of less dense material that were presumed to be portions of the cell's nuclear structure. 

### 3.3. Biochemical Tests

The MLO of mud crabs was able to ferment glucose, sucrose, lactose, and maltose without utilizing arginine and urea. The MLO grew in the absence of bovine serum and was not susceptible to digitonin. It was haemolytic for all three types of erythrocytes tested, but it did not haemadsorb these cells. No dye reduction occurred when the MLO was grown in MSM containing tetrazolium chloride or methylene blue. It grew in MLM containing a NaCl concentration from 0.5 to 3%.

### 3.4. Filtration Studies

Cultures were diluted 1 : 10 in MLM and then sequentially passed through membrane filters with 0.45 *μ*m and 0.22 *μ*m pore diameters. Filtration reduced the colony number from 2.35 × 10^7^ CFU mL^−1^ in the unfiltered culture to 9.00 × 10^6^ CFU mL^−1^ in the 0.45 *μ*m filtrate and to 6.59 × 10^4^ CFU mL^−1^ in the 0.22 *μ*m filtrate. 

### 3.5. Reversion Experiments

The isolate was diluted 1 : 10 in an MLM medium without antibiotics and incubated at 37°C for a total of eight passages. Each passage was subcultured on agar without antibiotics, and the cultures were examined for differences in colony morphology. No reversion was observed. 

### 3.6.  16S rRNA Gene Sequence Analysis

**Figure 3:**
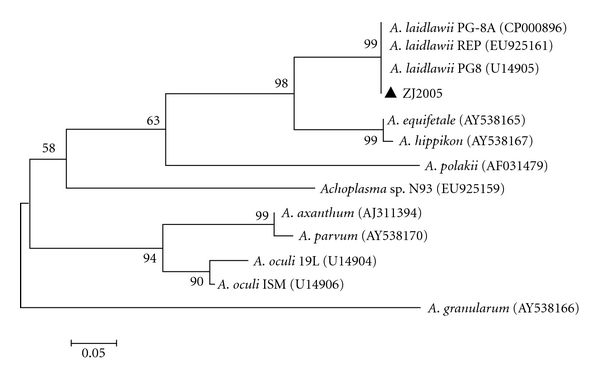
Phylogenetic tree based on 16S rRNA gene sequences showing the relationship of ZJ2005 and some members of the *Acholeplasma* group. Strain designations have been reported, and GenBank accession numbers are included. Bootstrap confidence level percentage values obtained from 1000 resamplings of the dataset are shown at the nodes. (bar, distance equivalent to 5 substitution per 100 nucleotides).

The 16S rRNA gene nucleotide sequence of ZJ2005 is 1425 nt in length (GenBank accession no. GU985440). Overall, the 16S rRNA gene nucleotide sequence similarity data placed strain ZJ2005 in the *Acholeplasma laidlawii* phylogenetic clade (Figure 3), where its closest relative (similarity score: 0.99) was an isolate provisionally named *A. laidlawii* PG-8A (GenBank accession no. FJ226559). 

### 3.7. Experimental Infections

Cumulative mortality by 15 days was 4/10 for Group 1 (1 on day 4, 1 on day 6, and 2 on day 7). For Group 2, mortality by 15 days was 3/10 (1 on day 8 and 2 on day 12). Interestingly, no clinical signs were observed in any of the dead experimental crabs, but MLOs were isolated from the gut and gill of all of the dead experimental crabs. No mortality, clinical signs, or MLOs were found in the unaffected experimental crabs and the crabs in control group. 

## 4. Discussion

The properties of the MLO isolated from mud crabs fulfilled the essential criteria for *Mollicutes* as proposed by the International Committee Systematic Bacteriology Subcommittee on Taxonomy (1995): it had a typical fried-egg colony form in culture, a polymorphic cell form, absence of a cell wall, passage through 0.45 *μ*m and 0.2 *μ*m filters, lack of reversion to bacteria, and resistance to ampicillin [1]. The results of 16S rRNA gene analysis and the biological, biochemical, and morphological studies indicated that the isolated MLO is a member of the genus *Acholeplasma*. Taxonomically, *Acholeplasma* belongs to the kingdom *Bacteria*, division *Firmicutes*, class *Mollicutes*, order *Acholeplasmatales*, family *Acholeplasmataceae*, and genus *Acholeplasma.* There are 15 recognized species in this genus, including saprotrophic and pathogenic species [27–30]. Although *Acholeplasma* spp. are widely distributed in nature and can be detected and isolated from different plant, avian, and mammalian sources [31–33], they have not been reported previously in aquatic animals. Our detection of *Acholeplasma* in *S. serrata* increases our knowledge about the host ranges of these organisms and should lead to further investigation of other possible aquatic hosts and to studies of possible relationships between terrestrial and aquatic hosts. 

The MLO in our study had 99% identity with *A. laidlawii* based on 16S rRNA genes. The three most useful criteria in *Acholeplasma* taxonomy are the 16S rRNA gene sequence, DNA-DNA hybridization analysis, and serology. The highest resolution is provided by 16S rRNA gene sequence analysis, which is useful for the discrimination of most species [9]. Our study has shown that the organism isolated from mud crabs is indeed a member of the genus *Acholeplasma*. However, further studies are needed to precisely identify the actual species. It is closely related to *A. laidlawii*, but it may represent a new species.

The MLO in the experimentally infected crabs did not cause high mortality or result in clinical signs of disease, which is not surprisingly because most *Acholeplasma* diseases are influenced by a variety of host and environmental factors. Moreover, a virulent strain can occur naturally, and some animals might carry *Acholeplasma* with no signs of disease until they are stressed [2]. However, the isolation of pure MLO from epithelium of gill and gut tissues of dead crabs suggests that the MLO might be only a cofactor for a reo-like virus, which was thought to be the main pathogen causing CD in mud crabs [34]. 

## References

[1] International Committee on Systematic Bacteriology Subcommittee on the Taxonomy of Mollicutes (1995). Revised minimum standards for the description of new species of the class *Mollicutes* (division *Tenericutes*). *International Journal of Systematic Bacteriology*.

[2] Simecka JW, Davis JK, Davidson MK, Ross SE, Stadtlander CT, Cassell GH, Maniloff J, McElhaney R, Finch L, Baseman J (1992). *Mycoplasma* diseases of animals. *Mycoplasmas: Molecular Biology and Pathogenesis*.

[3] Kirchhoff H, Beyene P, Fisher M (1987). *Mycoplasma mobile* sp. nov., a new species from fish. *International Journal of Systematic Bacteriology*.

[4] Ghadersohi A, Owens L (1999). Isolation, characterisation and DNA analysis of *Mycoplasma* spp. from moribund prawns *Penaeus monodon* cultured in Australia. *Diseases of Aquatic Organisms*.

[5] Krol RM, Hawkins WE, Overstreet RM (1991). Rickettsial and mollicute infections in hepatopancreatic cells of cultured Pacific white shrimp (*Penaeus vannamei*). *Journal of Invertebrate Pathology*.

[6] Yang JF, Wu YL (1992). Electron microscope study on mycoplasma-like of penaeid shrimp (*Penaeus chinensis*). *Donghai Marine Science*.

[7] Choi DL, Sohn SG, Park MA, Heo MS, Renault T (1996). Detection of a mollicute-like organism in kuruma shrimp, *Penaeus japonicus*. *Journal of Fish Pathology*.

[8] Frelier PF, Sis RF, Bell TA, Lewis DH (1992). Microscopic and ultrastructural studies of necrotizing hepatopancreatitis in Pacific white shrimp (*Penaeus vannamei*) cultured in Texas. *Veterinary Pathology*.

[9] Wang W, Wen B, Gasparich GE (2004). A spiroplasma associated with tremor disease in the Chinese mitten crab (*Eriocheir sinensis*). *Microbiology*.

[10] Harshbarger JC, Chang SC, Otto SV (1977). Chlamydiae (with phages), mycoplasmas, and rickettsiae in Chesapeake Bay bivalves. *Science*.

[11] Edgerton B, Owens L, Harris L, Thomas A, Wingfield M (1995). A health survey of farmed redclaw crayfish, *Cherax quadricarinatus* (Von Martens), in tropical Australia. *Freshwater Crayfish*.

[12] Jiménez R, Barniol R, Romero X, Machuca M (1998). A prokaryotic intracellular organism in the cuticular epithelium of cultured crayfish, *Cherax quadricarinatus (von Martens)*, in Ecuador. *Journal of Fish Diseases*.

[13] Zimmer RL, Woollacott RM (1983). Mycoplasma-like organisms: occurrence with the larvae and adults of a marine bryozoan. *Science*.

[14] Boyle PJ, Maki JS, Mitchell R (1987). Mollicute identified in novel association with aquatic invertebrate. *Current Microbiology*.

[15] Weng SP, Guo ZX, Sun JJ, Chan SM, He JG (2007). A reovirus disease in cultured mud crab, *Scylla serrata*, in southern China. *Journal of Fish Diseases*.

[16] Li YY, Xia XA, Wu QY, Liu WH, Lin YS (2008). Infection with *Hematodinium* sp. in mud crabs *Scylla serrata* cultured in low salinity water in southern China. *Diseases of Aquatic Organisms*.

[17] Timms L (1967). Isolation and identification of avian mycoplasma. *The Journal of Medical Laboratory Technology*.

[18] Aluotto B, Wittler RG, Willams CO, Faber JE (1970). Standardized bacteriologic techniques for the characterization of Mycoplasma species. *International Journal of Systematic Bacteriology*.

[19] Razin S, Cirillo VP, Razin S, Tully JG (1983). Sugar fermentation. *Methods in Mycoplasmology*.

[20] Barile MF, Razin S, Tully JG (1983). Arginine hydrolysis. *Methods in Mycoplasmology*.

[21] Razin S, Razin S, Tully TG (1983). Urea hydrolysis. *Methods in Mycoplasmology*.

[22] Tully JG, Razin S, Tully JG (1983). Tests for digitonin sensitivity and sterol requirement. *Methods in Mycoplasmology*.

[23] Gardelia RS, DelGiudice RA, Razin S, Tully JG (1983). Hemagglutination, hemadsorption, and hemolysis. *Methods in Mycoplasmology*.

[24] Kirchhoff H, Mohan K, Schmidt R (1997). *Mycoplasma crocodyli* sp. nov., a new species from crocodiles. *International Journal of Systematic Bacteriology*.

[25] Altschul SF, Gish W, Miller W, Myers EW, Lipman DJ (1990). Basic local alignment search tool. *Journal of Molecular Biology*.

[26] Tamura K, Dudley J, Nei M, Kumar S (2007). MEGA4: molecular Evolutionary Genetics Analysis (MEGA) software version 4.0. *Molecular Biology and Evolution*.

[27] Woese CR, Maniloff J, Zablen LB (1980). Phylogenetic analysis of the mycoplasmas. *Proceedings of the National Academy of Sciences of the United States of America*.

[28] Neimark H, London J (1982). Origins of the mycoplasmas: sterol-nonrequiring mycoplasmas evolved from streptococci. *Journal of Bacteriology*.

[29] Pollack JD, Banzon J, Donelson K (1996). Reduction of benzyl viologen distinguishes genera of the class *Mollicutes*. *International Journal of Systematic Bacteriology*.

[30] Angulo AF, Reijgers R, Brugman J (2000). *Acholeplasma vituli* sp. nov., from bovine serum and cell cultures. *International Journal of Systematic and Evolutionary Microbiology*.

[31] Tully JG, Krieg NR, Holt JG (1984). Acholeplasmataceae. *Bergey’s Manual of Systematic Bacteriology*.

[32] Razin S, Yogev D, Naot Y (1998). Molecular biology and pathogenicity of mycoplasmas. *Microbiology and Molecular Biology Reviews*.

[33] Ayling RD, Bashiruddin SE, Nicholas RAJ (2004). Mycoplasma species and related organisms isolated from ruminants in Britain between 1990 and 2000. *Veterinary Record*.

[34] Wadher BJ, Henderson CL, Miles RJ, Varsani H (1990). A mutant of *Mycoplasma mycoides subsp. mycoides* lacking the H_2_O_2_-producing enzyme L-*α*-glycerophosphate oxidase. *FEMS Microbiology Letters*.

